# Identification and Expression Analysis of Zebrafish Glypicans during Embryonic Development

**DOI:** 10.1371/journal.pone.0080824

**Published:** 2013-11-14

**Authors:** Mansi Gupta, Michael Brand

**Affiliations:** DFG- Center for Regenerative Therapies Dresden/ Cluster of Excellence (CRTD), Biotechnology Center, Technische Universität Dresden, Dresden, Saxony, Germany; Institute of Molecular and Cell Biology, Singapore

## Abstract

Heparan sulfate Proteoglycans (HSPG) are ubiquitous molecules with indispensable functions in various biological processes. Glypicans are a family of HSPG’s, characterized by a Gpi-anchor which directs them to the cell surface and/or extracellular matrix where they regulate growth factor signaling during development and disease. We report the identification and expression pattern of glypican genes from zebrafish. The zebrafish genome contains 10 glypican homologs, as opposed to six in mammals, which are highly conserved and are phylogenetically related to the mammalian genes. Some of the fish glypicans like Gpc1a, Gpc3, Gpc4, Gpc6a and Gpc6b show conserved synteny with their mammalian cognate genes. Many glypicans are expressed during the gastrulation stage, but their expression becomes more tissue specific and defined during somitogenesis stages, particularly in the developing central nervous system. Existence of multiple glypican orthologs in fish with diverse expression pattern suggests highly specialized and/or redundant function of these genes during embryonic development.

## Introduction

The members of the glypican family are extracellular matrix components which play essential roles in various biological processes. They are Heparan Sulfate Proteoglycans, composed of a cysteine-rich protein core to which heparan sulfate (HS) sugar chains are covalently attached at the C-terminal end. They usually mediate their function anchored on the exoplasmic cell membrane via a GPI-linkage, but can also be cleaved off the membrane and in some cases enter the circulation [1]. 

The HS sugar chains, being highly sulfated, attract a number of growth factors, due to their negative surface charge. Consequently, glypicans are known to modulate the activity of various growth factors like Wnt, Hedgehog (HH), Fibroblast growth factors (Fgfs) and BMP [2-5]. However not all functions of glypicans are mediated by their HS chains [6]. There are 6 glypican genes in humans (*GPC1-6*) and two in Drosophila (*dally* and *dally-like*), which code for the protein core [7]. Among glypicans, *dally* was the first mutant to be isolated in Drosophila displaying developmental defects in the eye, brain and wing [1]. This was attributed to a reduction in DPP signaling. Since then, various genetic and biochemical studies have associated glypicans to different developmental processes, growth and disease progression. They can function as low affinity co-receptors for growth factors or assist in their transport across cells [8]. In rodents, *Gpc1* regulates brain size via the modulation of Fgf signaling [4]. It is over-expressed in tumors from different organs [9] and was recently associated with the pathogenesis of a liver disorder, biliary artresia [10]. *GPC3* mutations in humans and mice result in Simpson-Golabi Behmel syndrome, characterized by pre- and postnatal skeletal anomalies and craniofacial malformations [11]. There is also a very high occurrence of GPC3 over-expression in hepatocellular carcinoma where it is considered as a serum marker and potential therapeutic target [12]. Gpc5 is a very specific enhancer of HH signaling and stabilizes the interaction between HH and its receptor Patched1 [3]. Gpc5 knockdown was seen in patients with neural tube defects [13] and this gene has also been implicated in nephrotic syndrome [14].

Among all glypicans, Gpc4 influences extremely diverse development processes. In *Xenopus* and zebrafish, Gpc4 regulates the convergent extension movements during gastrulation [2,15]. Its interaction with Fgf2 is also important for proper forebrain patterning in *Xenopus* [16]. Astrocytes secrete Gpc4 and Gpc6 which guide the formation of excitatory synapses [17]. Gpc4 also regulates insulin signaling via its interaction with the insulin receptor, and its levels in circulation correlate with an increased BMI [1]. Finally mutations in Gpc6 result in omodysplasia, characterized by shortened limbs and facial dysmorphism [18].

In contrast to humans and mice, the study of glypican function in zebrafish has so far been limited to *gpc3* and *gpc4*. A *gpc4* mutant in fish (*knypek*) displays defective convergent extension movements during gastrulation resulting in a reduced body length [2]. Gpc4 is directly involved in mediating non-canonical Wnt signaling in the embryos. Rescued *knypek* mutants also display defects in craniofacial cartilage development in larval and adult stages [19]. Gpc3 inhibits canonical Wnt β-catenin signaling after getting cleaved from the surface by Notum hydrolase and this regulation is also necessary for proper gastrulation [20]. 

We have previously found by single molecule analysis that extracellular matrix composition influences the mobility of Fgf8 forming a morphogen gradient in the extracellular matrix [21,22] Due to a lack of detailed information on fish glypicans as extracellular matrix components, we began to systematically characterize these genes. Firstly we identified and isolated 10 glypican genes from the fish. We studied their phylogeny with respect to humans and analyzed their expression pattern at various stages of embryonic development. Our findings indicate that glypicans are overall conserved between mammals and zebrafish, and may serve both generalized and highly tissue-specific functions in developing tissues.

## Materials and Methods

### Ethics Statement

All animal experiments were carried out in strict accordance with European Union and German laws (Tierschutzgesetz). All experimental procedures were approved by the animal ethics committee of the TU Dresden and the Landesdirektion Sachsen (approval number: AZ 24D-9168.11-1/2008-4). This institutional review board specifically approved this study.

### Zebrafish husbandry

Zebrafish were raised and maintained as described previously [23]. The wild-type line used was TL. Zebrafish embryos were obtained by natural spawning of adult fish and staged according to hours post fertilization (hpf) or standard criteria [24]

### Bioinformatics analysis

Ensembl Zv9, GenBank and DFCI EST databases were used to identify zebrafish glypican sequences. Human glypican sequences were blasted against all three databases and the obtained fish sequences were confirmed for the presence of the glypican domain. Mega5.1 software was used for phylogenetic analysis. ClustalW and the Jalview software were used for generating and viewing the multiple sequence alignment, respectively. Protein sequence similarity and identities were obtained using NCBI BLASTp. Cinteny server (http://cinteny.cchmc.org/) and genomicus server were used for syntenic analysis.

### Molecular Cloning

Zebrafish mRNA was isolated from 24 and 48 hpf old embryos using the Trizol/Phenol-Chloroform method. cDNA was prepared from RNA using SuperScriptIII First-Strand Synthesis system (Invitrogen). The open reading frames of glypican genes were cloned into Topo vector using the primers given in Table 1. *gpc2* full length sequence was obtained by performing RACE using SMARTer RACE cDNA amplification kit (Clontech) with primer: CAGCCCTGAAACACCTTAGCAGAGA for 5’ RACE and primer: AGACGCGCGGCAGGTACCTGCCAGCAG for 3’ RACE.

**Table 1:** Primer list.

Gene Name	Forward Primer	Reverse Primer
Primers for cloning		
*gpc1a*	ATGGATCTGACAGCGGTCGC	TGATCTAGATTATCGTCTGAGCAATAGACTC
*gpc1b*	ATGGGTTTTGTCTCGCTGGT	GCATCTAGATTATCGCCTAAGCAAGACTGT
*gpc2*	ATGAAGATGATGAAGGTGGTGATGAAGAT	TTACAGACACAGACAGAGAGTAAAGC
*gpc3*	ATGATGCCTGGACTGAAGTTG	AGCTCGAGATCACTGAAGACCCAGTGTTATG
*gpc4*	CCTGGATCCATGAAGATGATCGTTGTGTT	GTGCTCGAGTTATCTTGTTTGGAGAGTGA
*gpc5a*	ACGGGATCCATGTCTCTTCACCTAAAATC	GTCTCGAGTTCATTGTGTCTCCTTACTGG
*gpc5b*	AATGCTCCGCGGGACAGCA	TACTCGAGATCAAGGCCACAGGAGGAGCT
*gpc5c*	CGAATTCATGTCACGCGTGAATGTCAGCT	TCTCGAGTTCGACCCTTAGAGCTAAGGTATG
*gpc6a*	TAGGATCCATGGTGAAGACACCTGTCGT	CACTCGAGTTATCTCCAGCAGAGCAGCA
*gpc6b*	AAGGATCCATGTGGGCTGTGTGCGCGCT	GTCTCGAGCTATCTGCAGGTCAGTGCCA
Primers for RT-PCR		
*gpc2*	CGA AGC TGC GCG GAG TCC CG	ACCAGTTGGTCCAGGTCCGTCC
*gpc3*	AGTGCGCTCATCCTTTCAGT	CATACGCAGAGCTCCCTTTC
*gpc5a*	GGATACTGCCTGAACGTGGT	TTTTCAGCCTGCGCTTTAAT
*gpc5b*	TCTACCTTCGAGGCGATGAT	CTCGCATCACGTTGAGACAT
*gpc6a*	CACCAAAGGCTTCAGTCTCC	CCATAAGTCCCTGGACGAAG
*gpc6b*	ACGTCAACCTGGAGGAGATG	ATGGGCTCCATCACAGACTC

### RT-PCR

cDNA was prepared from different developmental stages as mentioned above and used for RT-PCR. Full length primers (Table 1) were used to amplify *gpc1a*, *gpc1b*, *gpc4* and *gpc5c*. The primers used for *gpc2*, *gpc3, gpc5a*, *gpc5b*, *gpc6a* and *gpc6b* are also mentioned in Table 1. The PCR reaction was carried out using DreamTaq DNA polymerase (Fermentas) for 30-35 cycles.

### In Situ Hybridization

Embryos at the desired stage were fixed in 4% PFA and *in situ* hybridization was carried out according to the protocol described previously [25]. Full-length antisense probes were synthesized using T7/SP6 polymerase, Digoxigenin label (Roche) and linear Topo vectors. The color was developed using BM purple and used for imaging. Flat mounts were prepared after removing yolk sac and mounted in glycerol.

## Results

### Identification of Fish Glypicans

There are 6 glypican genes in the human genome (*GPC1-6*). These sequences were blasted against Ensembl Zv9, GenBank and DFCI zebrafish EST databases to obtain the corresponding zebrafish glypicans. A previous study also reported 6 fish glypicans [10] but we identified 10 genes and named them according to their similarity to the human orthologs. The accession numbers of all genes from the different databases are shown in Table 2. New GenBank accession numbers were obtained for most sequences. Also indicated are the corresponding human orthologs and their peptide length. 

**Table 2:** Accession numbers of zebrafish glypicans.

**Gene Name**	**Chromosome No.**	**Ensembl Gene ID**	**GenBank Acc No. (old)**	**DFCI Identifier**	**GenBank Acc. No. (new)**	**Peptide length (a.a.)**	**Human homolog/Peptide length**	**Comments**
*gpc1a*	22	ENSDARG00000019341*	BC109411	TC366647	KC836776	554	*HsGPC1*/558	Gpc1^**^
*gpc1b*	2	ENSDARG00000090456	BC053161*	TC368439	KC836777	541	*HsGPC1*/558	
*gpc2*	14	ENSDARG00000037150	BC133103*	TC411825	KC791423	576	*HsGPC2*/579	Identified by 5’ and 3’ RACE
*gpc3*	14	ENSDARG00000032199	XM_682922*	TC371608	-	590	*HsGPC3*/603	[31]
*gpc4*	14	ENSDARG00000015472*	NM_131860	TC419247	-	557	*HsGPC4*/556	[2]
*gpc5a*	Zv9_NA	ENSDARG00000088858	XM_001920514	TC414869*	KC791424	582	*HsGPC5*/572	
*gpc5b*	22	ENSDARG00000024588*	--	TC376562	KC791425	610	*HsGPC5*/572	Gpc5^**^
*gpc5c*	2	ENSDARG00000074082*	--	--	KC999393	523	*HsGPC5*/572	
*gpc6a*	12 1	ENSDARG00000091739 ENSDARG00000086960	BC151902* (complete) XM_003197610	TC370142	KC999394	562	*HsGPC6*/555	
*gpc6b*	9	ENSDARG00000036468	XM_003199260*	NP13316404	KC999395	543	*HsGPC6*/555	Gpc6^**^

* cloned sequences; *HsGPC*: Human glypican

** Previously reported names [10]

**Figure 1:**
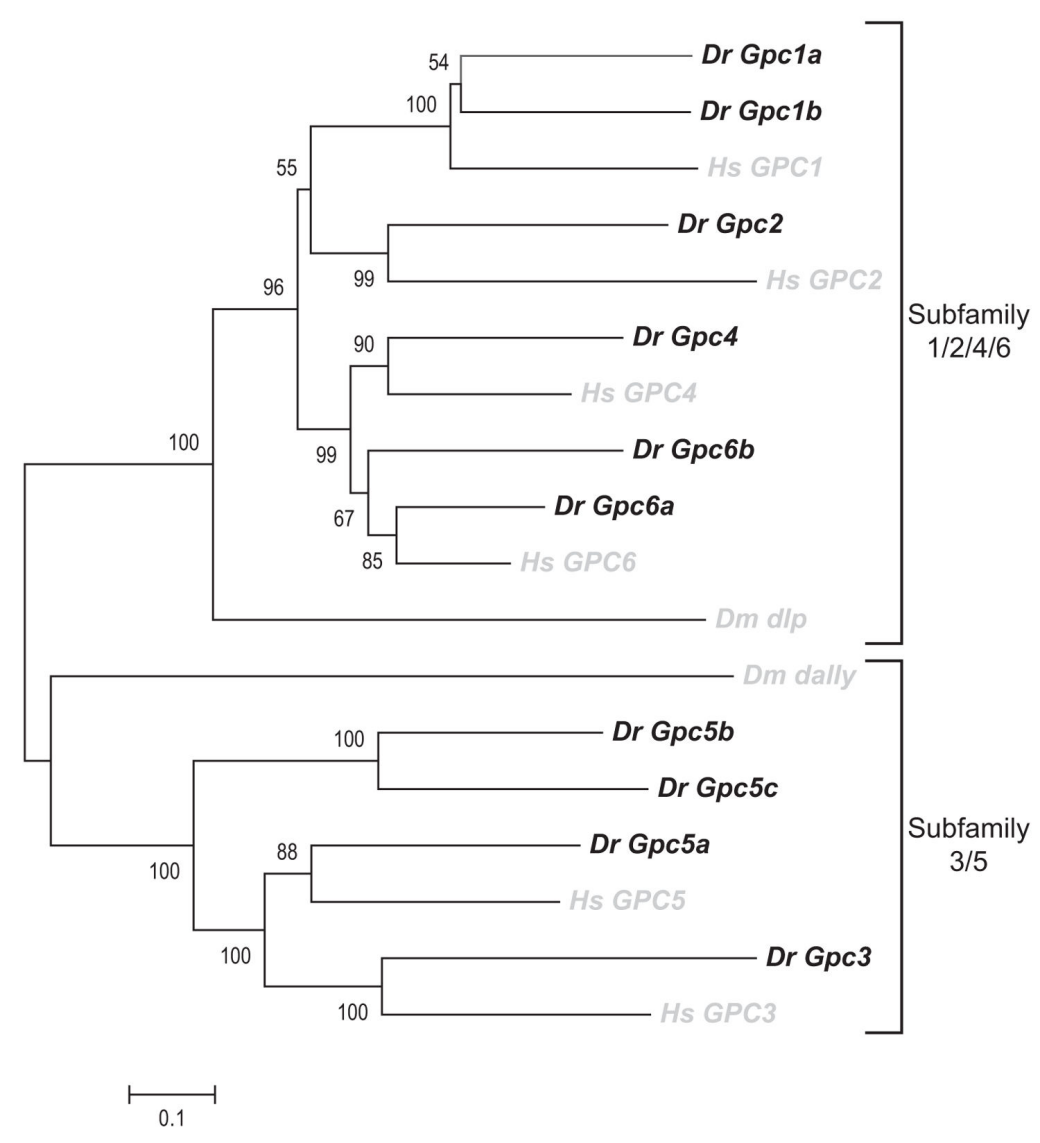
Phylogenetic tree of glypican genes from Danio rerio, Homo sapiens and Drosophila melanogastor. Phylogenetic comparison was carried out for glypicans genes sequences from zebrafish (Dr in black), humans and Drosophila (Hs and Dm in grey). The evolutionary tree was constructed using Neighbor-Joining method in the MEGA5.1 software using the Poisson model and partially deleted dataset. 500 bootstrap replications were used as a test of phylogeny and the values are indicated next to the branch. Branch length corresponds to evolutionary distances which denote the number of amino acid substitutions per site. Scale bar: 0.1

Glypican gene family members can be grouped into two sub-families, as reported previously [7]: *GPC1/2/4/6* and *GPC3/5* family (Figure 1). Phylogenetic comparison of fish, human and *Drosophila* sequences revealed that all fish genes cluster with their human orthologs. The two genes in Drosophila, *dally* and *dally-like protein* each belong to a different family. Multiple orthologs of human genes are commonly found in fish genome due to a duplication event which occurred before the radiation of teleosts [26]. Corresponding to *HsGPC2* we identified one new ortholog in the fish genome (*Drgpc2*). Two new orthologs were identified corresponding to *HsGPC1* (*Drgpc1a* and *Drgpc1b*) and *HsGPC6* (*Drgpc6a* and *Drgpc6b*) each, and 3 corresponding to *HsGPC5* (*Drgpc5a*, *Drgpc5b* and *Drgpc5c*). All glypican proteins exhibit more than 56% sequence similarity with their corresponding human orthologs (Table 3).

**Table 3:** Protein sequence similarity.

	Identity %	Similarty %
DrGpc1a vs HsGPC1	54	70
DrGpc1b vs HsGPC1	52	68
DrGpc2 vs HsGPC2	46	63
DrGpc3 vs HsGPC3	43	58
DrGpc4 vs HsGPC4	58	72
DrGpc5a vs HsGPC5	55	73
DrGpc5b vs HsGPC5	39	56
DrGpc5c vs HsGPC5	39	59
DrGpc6a vs HsGPC6	68	81
DrGpc6b vs HsGPC6	60	77

**Figure 2:**
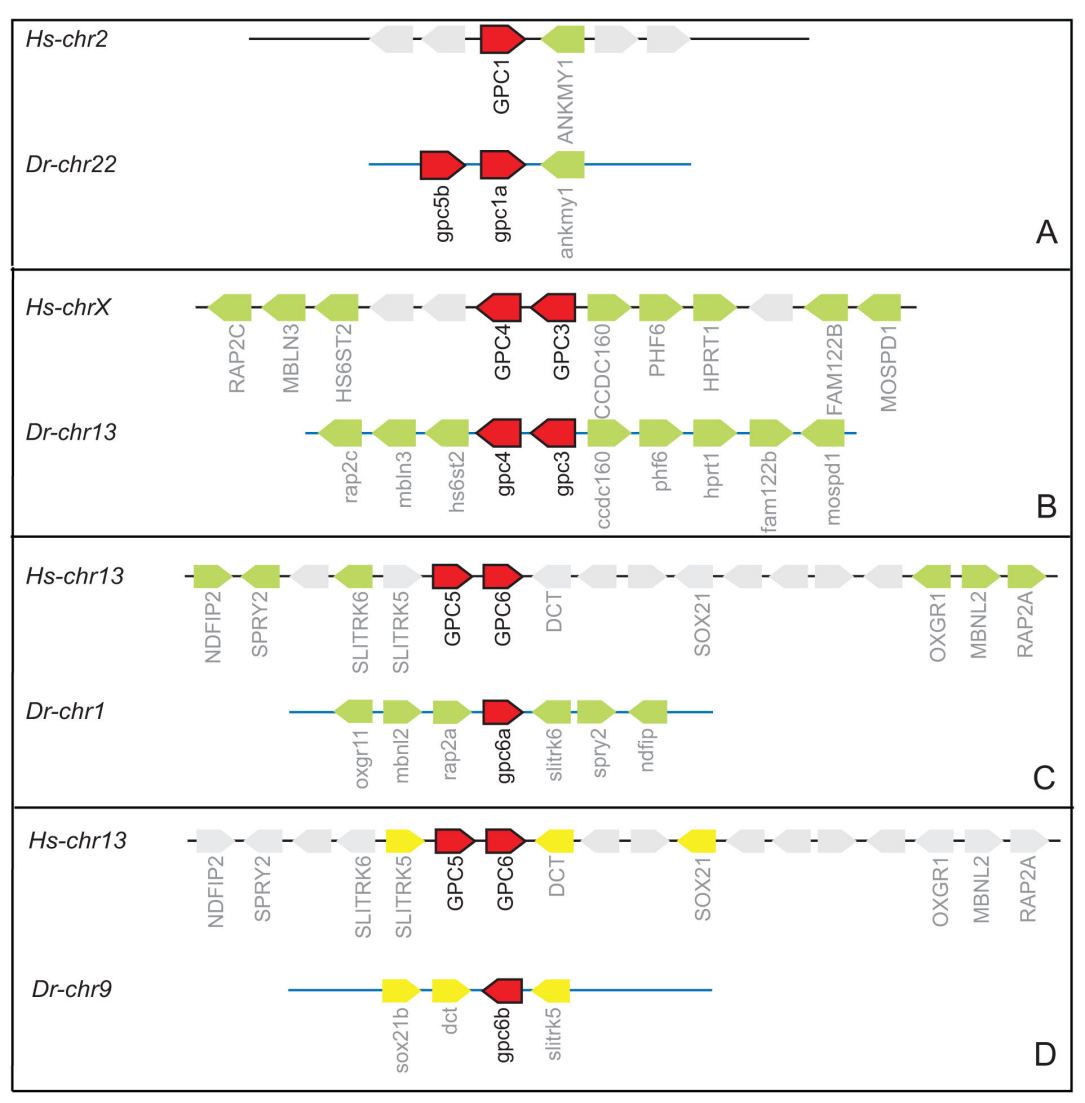
Syntenic conservation between zebrafish and humans glypicans. The figure depicts arrangement of genes surrounding glypicans on corresponding chromosomes in humans and zebrafish. Chromosome numbers from humans (*Hs*) and zebrafish (*Dr*) are indicated on the left. Glypican genes are represented in red and conserved markers around glypicans are in green or yellow. Non-conserved genes are in grey. The diagram is not to scale.

In order to identify true orthologs of *HsGPC1*, *HsGPC5* and *HsGPC6*, we investigated the chromosomal syntenic relationship between fish and human glypican genes by comparing common markers flanking these genes. The gene cluster containing *GPC3* and *GPC4* is well conserved in Eumetazoa and is also seen in zebrafish (Figure 2B) [7]. Of the two *gpc1* in fish, *gpc1a* shares one adjacent marker with the *HsGPC1* (Figure 2A), although both *Drgpc1a* and *Drgpc1b* share an almost similar amino acid sequence identity with *HsGPC1* (Table 3). This indicates that *Drgpc1a* is likely to be a functional ortholog of *HsGPC1*. Fish has two orthologs for *HsGPC6*, namely *Drgpc6a* and *Drgpc6b*. Both genes share some synteny with the human form, such that certain markers are common between *HsGPC6* and *Drgpc6a* and others are common between *HsGPC6* and *Drgpc6b* (Figure 2C and 2D). This suggests that both zebrafish genes are derived from the same ancestral locus but have undergone rearrangements resulting in the final gene arrangements. Currently, *Drgpc6a* is annotated on two chromosomes, Chr1 and Chr12 (Ensembl Zv9), but only the Chr12 version is syntenic with *HsGPC6*. 

In the human genome, *GPC5* and *GPC6* are present adjacently, but this contiguity is lost in the fish genome. There are 3 copies of *gpc5* in fish and *Drgpc5a* has the maximum amino acid sequence identity to *HsGPC5* (Table 3) but none are syntenic with humans. On fish Chr22, *Drgpc5b* and *Drgpc1a* are present contiguously, an arrangement not seen in mammals. Absence of synteny implies that along with whole genome duplication, individual gene duplications for fish *gpc5* might have resulted in the observed multiple paralogs. 

### Glypican conserved domain structure

**Figure 3:**
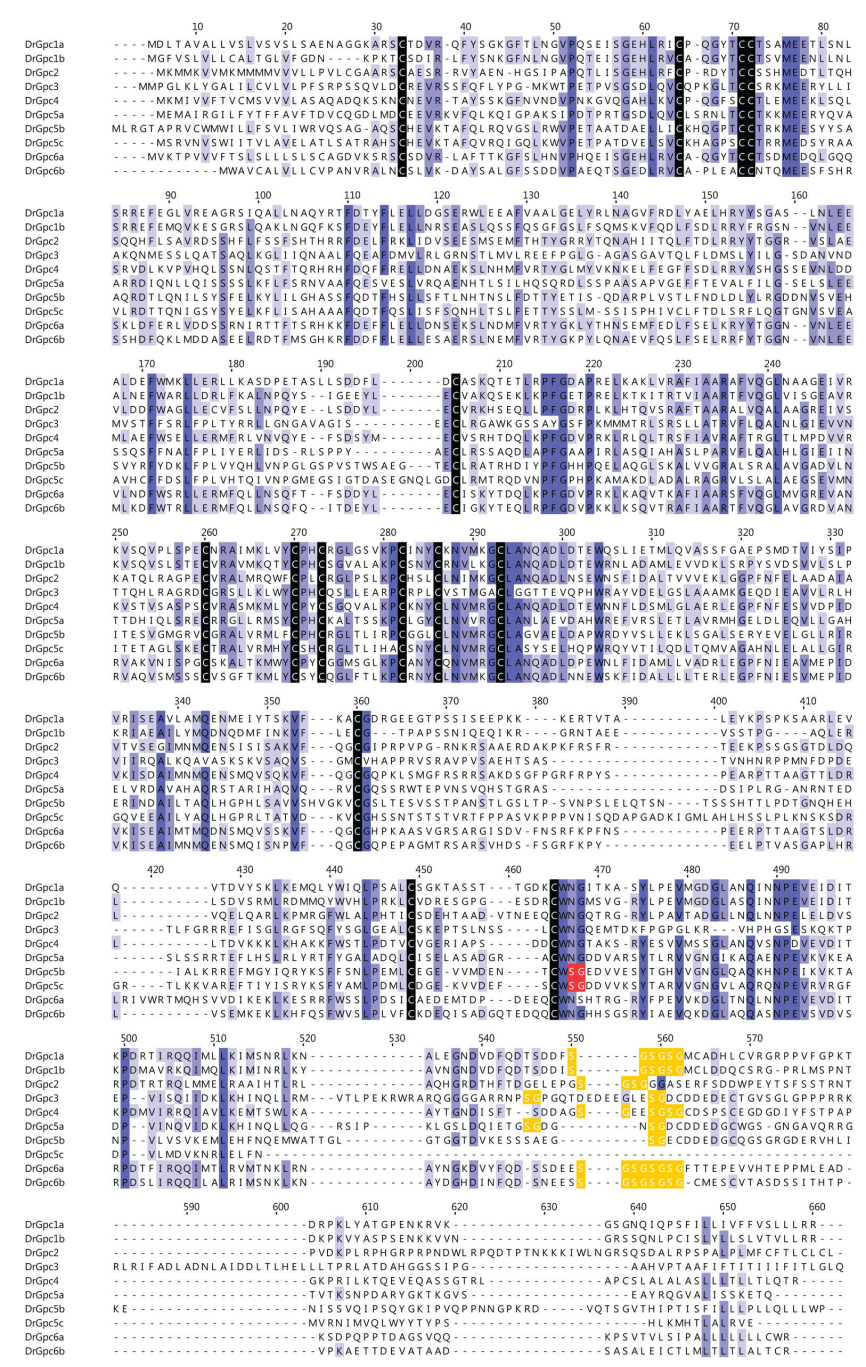
Multiple sequence alignment of glypican protein sequences from zebrafish. All protein sequences were aligned to compare conserved features between glypicans. Residues highlighted in blue are well conserved. Black highlights indicate the 14 conserved cysteine residues; yellow highlights are the multiple Ser-Gly repeats flanked by acidic residues for sugar attachment; in red is the potential chondroitin sulfate attachment site.

The glypican family of proteins is characterized by a large and highly conserved N-terminal glypican domain and a C-terminal region containing the residues for heparan sulfate and Gpi-anchor attachment. Multiple sequence alignment confirmed the presence of 14 conserved cysteine residues in all zebrafish glypicans (Figure 3). All these residues are involved in disulfide linkages and are necessary to maintain the folded structure [27]. The HS attachment site is always present within 50 residues of the C-terminal placing them very close to the plasma membrane [28]. It consists of repeating Ser-Gly (SG) cluster (n≥2) flanked by acidic residues on both sides [29]. There are 4 SG repeats in Gpc1a, Gpc1b, Gpc6a and Gpc6b; 3 repeats in Gpc4; 2 repeats in Gpc2, Gpc3, Gpc5a and 1 repeat in Gpc5b. Gpc5c and also Gpc5b contain a separate SG dipeptide followed by acidic amino acids, a potential chondroitin sulfate priming motif. Since HsGpc5 is known to carry both heparan sulfate and chondroitin sulfate chains [30], this feature may be conserved even in zebrafish Gpc5. 

### Spatiotemporal expression of glypicans during early development

**Figure 4:**
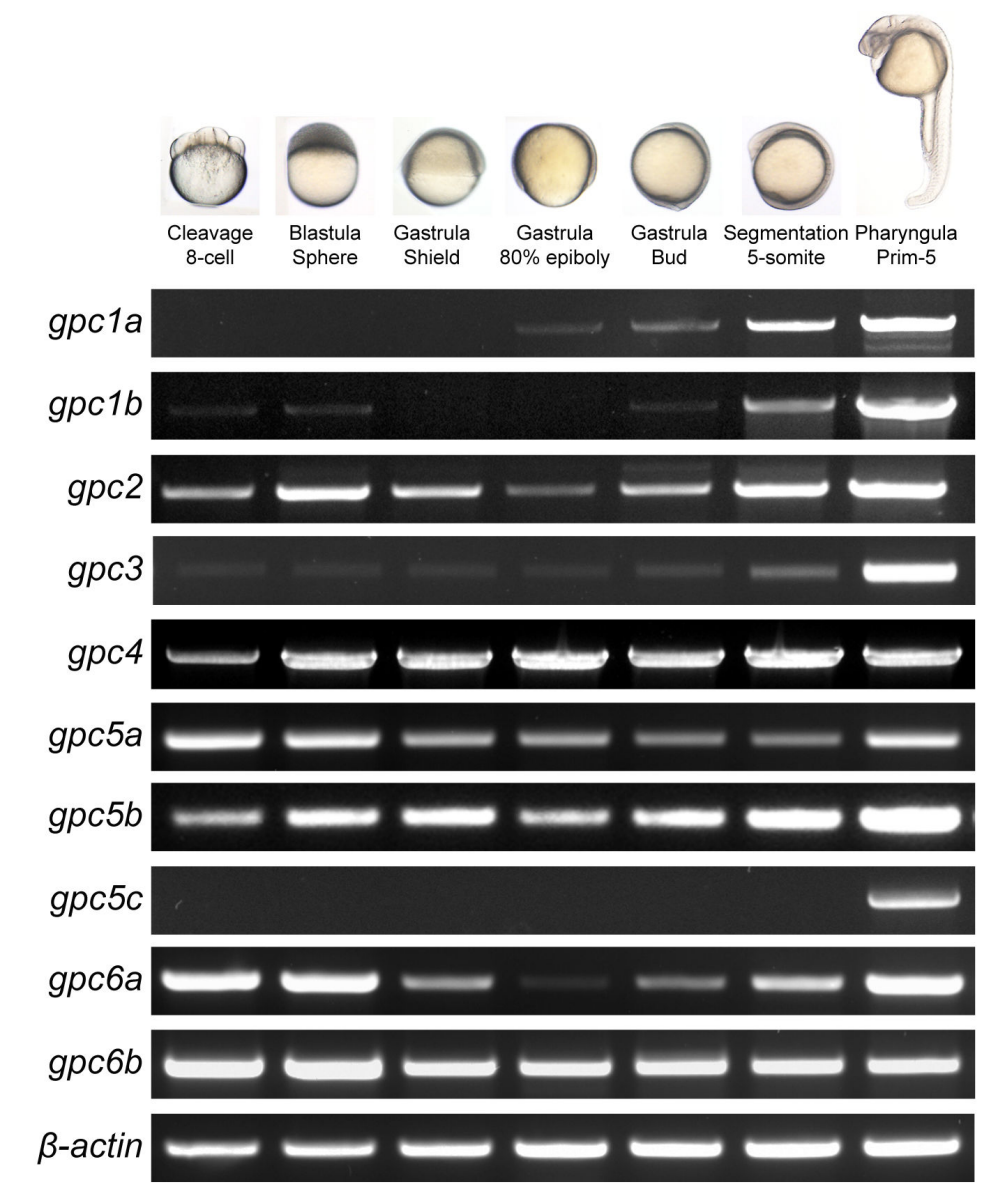
Temporal mRNA expression of glypicans genes during early embryonic development. RT-PCR was performed for all 10 genes using cDNA from specified stages of development. *β-actin* was used as a control. Ep, epiboly; som, somites; hpf, hours post fertilization.

**Figure 5:**
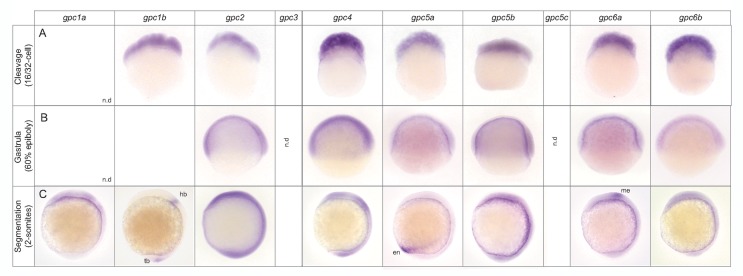
Spatial expression of zebrafish glypicans during cleavage to early segmentation stages. *In* *situ* hybridization was performed to detect glypican mRNA during early developmental stages. Lateral views with anterior to the top (A, B, C) and dorsal to the right (B, C). Developmental stages and gene names are indicated. tb, tailbud; hb, hindbrain; en, endoderm; me, mesencephalon; n.d: not detected.

**Figure 6:**
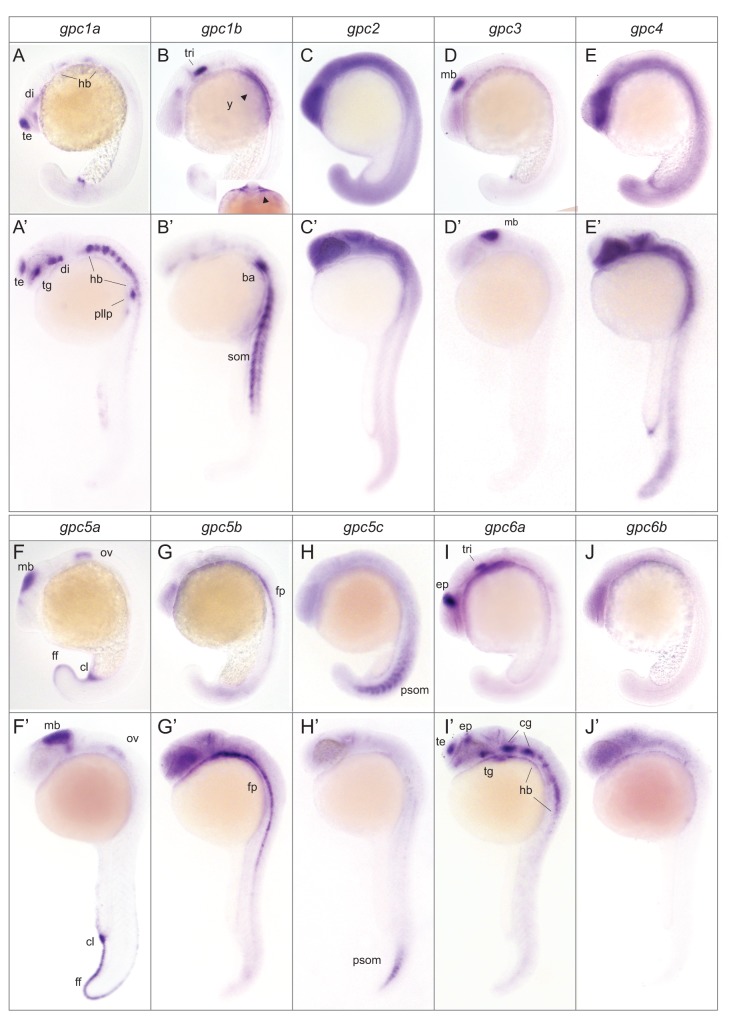
Spatial expression pattern of zebrafish glypicans during segmentation and pharyngula stages. *In* *situ* hybridization was carried out to detect glypican mRNA at 20 somite stage: 19 hpf and pharyngula stages: 24-26 hpf. Lateral view of whole mount embryos, anterior to the left. Expression pattern of *gpc1a* (A, A’), *gpc1b* (B, B’); subfigure in B is an optical section indicating expression in yolk cells (arrowheads) and branchial arch primordium, *gpc2* (C, C’), *gpc3* (D, D’), *gpc4* (E, E’), *gpc5a* (F, F’), *gpc5b* (G, G’), *gpc5c* (H, H’), *gpc6a* (I, I’), *gpc6b* (J, J’). di, diencephalon; te, telencephalon; hb, hindbrain; tg, tegmentum; nc, neural crest; pm, paraxial mesoderm; som, somites; ba, branchial arch; y, yolk; mb, midbrain; ff, fin fold; ov, otic vesicle; fp, floor plate; psom, posterior somites; ep, epiphysis; tri, trigeminal placode; cl, cloaca; pllp, posterior lateral line primordium; cg, cranial ganglion.

**Figure 7:**
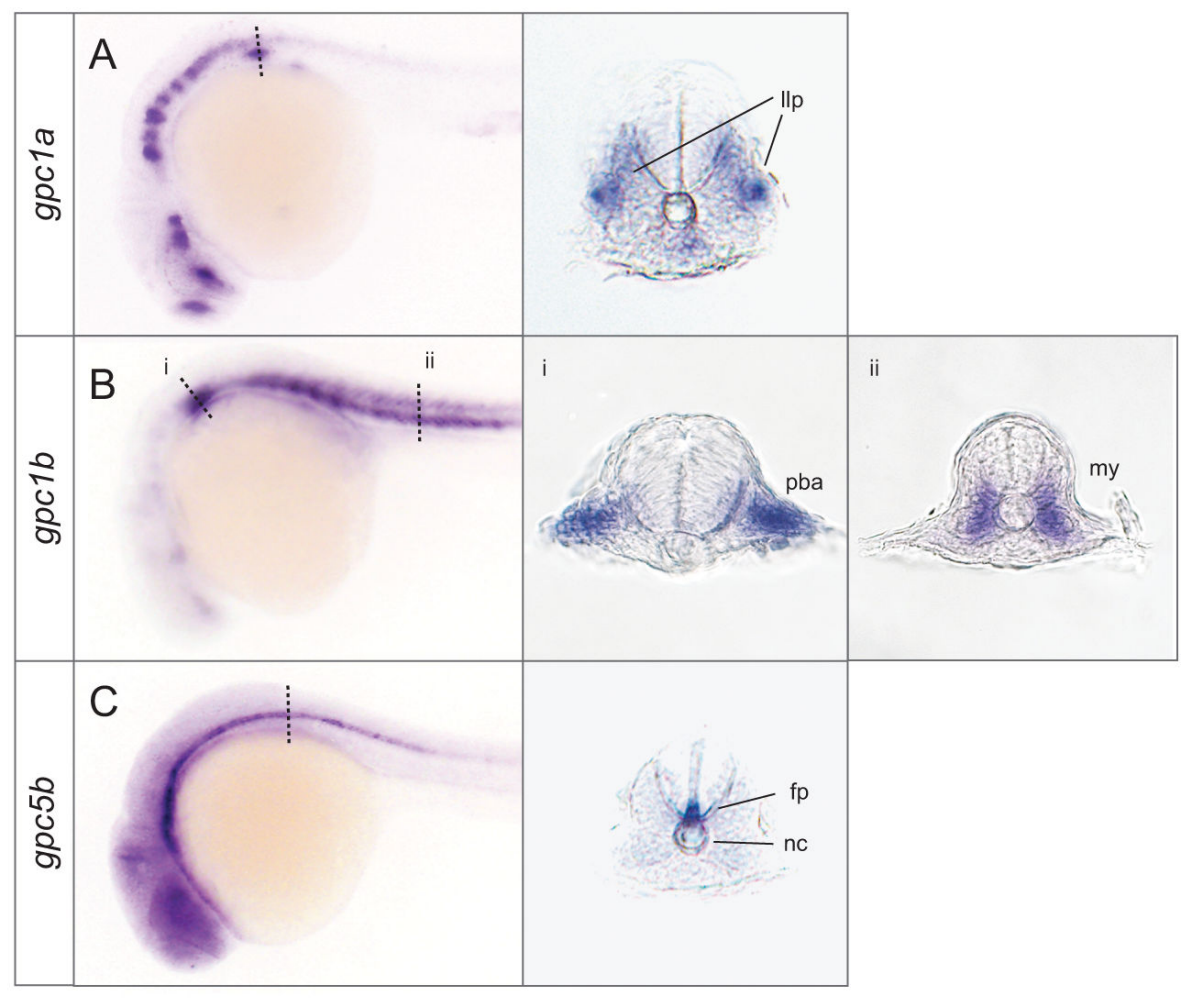
Specific expression domains for *gpc1a*, *gpc1b* and *gpc5b*. Transverse cross sections revealed the expression details of *gpc1a*, *gpc1b* and *gpc5b*. Dotted line indicates the plane of section. pllp: posterior lateral line primordium; pba: posterior branchial arch; my: myotome; fp: floor plate; nc: notochord.

The composition of extracellular matrix differs considerably between specific tissues and stages of development. A previous study has focused on the expression of fish glypicans at 5 day post fertilization only in the developing liver [10]. We now analyze the temporal and spatial expression of all glypicans from cleavage to pharyngula stages using Reverse transcriptase PCR (RT-PCR) and *in situ* hybridization (ISH) (Figures 4-7). As reported before, *gpc4* mRNA is present at all stages of embryonic development [2]. A constant expression (both maternal and zygotic) was also seen for *gpc2, gpc3*, *gpc5a*, *gpc5b*, *gpc6a* and *gpc6b*. Maternal expression was detected for *gpc1b*, but zygotic expression started only during segmentation.. Maternal expression was not seen for *gpc1a* and *gpc5c*. *gpc1a* transcripts were first detected at gastrulation stage and gradually increased during segmentation period and *gpc5c* was detected from late segmentation stages. 

These findings were confirmed and extended by whole mount *in situ* hybridization. As seen by RT-PCR, maternal contribution was observed for *gpc2*, *gpc4, gpc5a*, *gpc5b*, *gpc6a* and *gpc6b* (Figure 5A). *gpc1b* was also detected by ISH at cleavage stages but not later during gastrulation. *gpc3* transcripts could not be detected either during blastula or gastrula stages by ISH probably due to low levels of transcripts.

Gastrulation marks the process when cell movements result in the formation of the germ layers and embryonic axis. Gpc3 and Gpc4 influence this process in a mutually exclusive manner [20]. Along with *gpc4*, we also detected *gpc2*, *gpc5a*, *gpc5b*, *gpc6a* and *gpc6b* transcripts at 60% epiboly (Figure 5B). Further functional studies will uncover whether all glypican genes function independently or exhibit redundancy with each other during early stages. 

We next examined the expression of glypicans during the segmentation period. Beginning from the 2-somite stage, the expression domains of glypicans became more tissue-restricted (Figure 5C). Whereas *gpc2*, g*pc5b* and *gpc6b* were seen ubiquitously on the dorsal side of the embryo, g*pc1a*, *gpc1b* and *gpc5a* showed restricted expression domains in the developing nervous system. *gpc1a* expression was seen throughout the brain primordium, *gpc1b* was present near the hindbrain and tailbud region and *gpc6a* in the mesencephalic region. *gpc5a* was selectively expressed in the ventral endoderm. *gpc3* or *gpc5c* were not detected.

By the 20 somite stage, all glypicans were detected by ISH. Most genes were expressed in a variety of neural tissues. *gpc1a* was detected in the primordial telencephalon, diencephalon and hindbrain (Figure 6A). By the 24 hpf stage, its expression became more confined to these regions and was also detected in the posterior lateral line primordium (Figure 6A’, Figure 7A). g*pc1b* was initially seen in the trigeminal placode and the yolk cells during segmentation (Figure 6B) and at 24 hpf, in somites (Figure 6B’) and the branchial arch (Figure 7B). 

*gpc3* was expressed very specifically in the midbrain, coinciding with the time of active patterning processes in this tissue (Figure 6D, 6D’). This is in contrast to previous observation where g*pc3* was suggested to be restricted to the prospective hindbrain [31]. *gpc4* was expressed broadly throughout the embryo but excluded from most dorsal regions, including the telencephalon (Figure 6E. E’). g*pc5a* was detected in the midbrain, otic vesicle, primordial fin fold and cloaca (Figure 6F) and this pattern persisted at 24 hpf (Figure 6F’). *gpc5b* was restricted to the floor plate from the 20 somite stage (Figure 6G), but at 24 hpf, its expression was additionally detected throughout the nervous system (Figures 6G’, 7C)

*gpc6a* was initially very strongly expressed in the epiphysis and the trigeminal placode (Figure 6I) and at 24 hpf, it was also seen in the telencephalon, tegmentum, cranial ganglia and the hindbrain (Figure 6I’). *gpc6b* was present uniformly in the nervous system (Figure 6J, 6J’). 

*gpc2* had the most widespread expression domain, seen ubiquitously in the embryo (Figure 6C, 6C’). In contrast, *gpc5c* was expressed outside the nervous tissue, in posterior somites (Figure 6H, 6H’). 

## Discussion

A previous study of glypican expression pattern in mice revealed that these genes are expressed predominantly in the embryonic brain [32]. In support of this, we also observed widespread expression of zebrafish glypicans in the developing nervous system. Apart from *gpc5c*, which is found in the posterior somites, all zebrafish genes are present either in very specific domains or more generally in the brain. Hence glypicans might have a conserved function during the development and patterning of the nervous system. Several studies in mammals have revealed the importance of glypicans in brain development [4,13] . In *Xenopus* also, Gpc4 is required for proper patterning of the forebrain [16]. Interestingly, the zebrafish *gpc4* mutant, *knypek*, displays craniofacial skeletal defects during the larval to adult stages due to improper chondrocyte and cartilage growth ([33].

A direct comparison of expression domains between mouse versus zebrafish glypicans reveals interesting analogous features. During rat and mouse embryogenesis, *Gpc1* is predominantly expressed in the developing nervous system and skeletal system. It is specifically present in the proliferating neural progenitors of forebrain, midbrain and hindbrain at E14/ E18 pharyngula stages [34]. The expression of zebrafish *gpc1a* seen at 24 hpf corresponds well with that of rat/mouse *Gpc1*. Moreover we observed partial synteny between human *GPC1* and zebrafish *gpc1a* (Figure 2A). Hence based on comparable expression pattern and partial synteny, we conclude that zebrafish *gpc1a* is more likely to be the true ortholog of mammalian *Gpc1*.

In a previous study on *gpc3*, it was reported that it is ubiquitously present during early zebrafish development [31]. We detected transcripts of *gpc3* by RT-PCR at all stages, but these could not be detected by ISH, possibly due to very low levels. Interestingly, *gpc3* expression in zebrafish does not correlate with mammalian expression. *GPC3* is the causative gene for Simpson-Golabi Behmel Syndrome and is strongly up-regulated in hepatocellular carcinoma. In humans and mice, it is expressed in hepatocytes, several kidney structures, mesenchyme of limb buds, vertebrae and the liver [35]. In contrast, we observed a very specific expression of zebrafish *gpc3* only in the midbrain. It is likely that this function of *Gpc3* is preserved only in mammals, although later developmental stages in zebrafish remain to be analyzed. 

Another parallel can be drawn between the *Gpc5* expression patterns. In *Xenopus*, *gpc5* is expressed strongly during neurulation and becomes restricted to the floor plate, somites and optic vesicle in the late neural stages [13]. It has been implicated in neural tube closure defects [13]. Zebrafish *gpc5b* is also specific to the floor plate and is diffusely present in the nervous system. This indicates that *gpc5b* might be the cognate zebrafish gene and also suggests for a potential conserved role of *gpc5b* in neural tube defects. In mice and humans, *Gpc5* is prominent in kidney, limb and brain both in developing and adult tissues [30]. But so far, we did not examine later stages of zebrafish development for *gpc5* expression.

Taken together, zebrafish glypicans exhibit quite dynamic expression pattern during early embryonic development. The 10 genes identified in this study are selectively turned on in different tissues and hence have the potential to mediate important morphogenetic functions either independently or in conjunction with each other. Multiple signaling pathways are known to be modulated by glypicans in a context dependent manner [2-5], and our data will therefore contribute to elucidating the mechanisms by which glypicans mediate these functions. 
